# ACE2-PNA conjugates exploit viral endocytosis for targeted intracellular delivery and exhibit dual antiviral efficacy against SARS-CoV-2

**DOI:** 10.1186/s43556-026-00520-6

**Published:** 2026-08-03

**Authors:** Yifei Wang, Jinghan Xu, Yiquan Chen, Xin Xiao, Yiru Zhu, Liyang Yu, Wen Shi, Jingchao Li, Jianhua Li, Chenggang Zhu

**Affiliations:** 1 College of Life Sciences, Zhejiang University, Hangzhou, China; 2 Zhejiang Key Laboratory of Public Health Detection and Pathogenesis Research, Department of Microbiology, Zhejiang Provincial Center for Disease Control and Prevention, Hangzhou, China; 3 School of Medical Technology and Information Engineering, Zhejiang Chinese Medical University, Hangzhou, China

**Keywords:** SARS-CoV-2, Peptide nucleic acids, Angiotensin-converting enzyme 2, Drug delivery systems

## Abstract

The COVID-19 pandemic and its protracted consequences underscore the urgent need for more effective antiviral strategies. Current antiviral strategies face a persistent challenge achieving sufficient viral suppression while minimizing off-target toxicity, particularly against such highly mutable viruses. Here, we describe a Receptor-Drug Conjugate (RDC) strategy, in which a therapeutic payload is covalently linked to a decoy receptor, enabling virus-triggered targeted intracellular delivery. Angiotensin-converting enzyme 2 (ACE2), as the essential receptor for SARS-CoV-2 entry, has been widely exploited for virus-neutralizing strategies. Targeting SARS-CoV-2 as a proof-of-concept, we conjugated peptide nucleic acids (PNAs) designed to target the viral *ORF1ab* region (±30 bp) to ACE2-Fc. The ACE2-PNA conjugate demonstrated superior inhibitory efficacy against multiple SARS-CoV-2 variants relative to ACE2-Fc alone. We confirmed that ACE2-PNA retains the extracellular neutralization activity of soluble ACE2, while being selectively internalized into virus-infected cells via virus-mediated endocytosis. Owing to its inherent protease resistance, the PNA component remains intact upon cytoplasmic entry and subsequently exerts antisense inhibitory activity against viral RNA. The mechanistic feasibility of ACE2-PNA was further validated in a mouse model. Collectively, RDC represents a novel virus-triggered targeted delivery platform that confers dual antiviral efficacy through extracellular virion neutralization and intracellular inhibition of viral replication. It has significant implications for reducing off-target toxicity and enhancing antiviral potency, and is furthermore readily adaptable to diverse viral pathogens and therapeutic payloads.

**Supplementary Information:**

The online version contains supplementary material available at 10.1186/s43556-026-00520-6.

## Introduction

In recent years, a growing number of viruses have posed significant threats to global public health through cross-species transmission and climate change-driven expansion. To date, over 200 viruses are known to infect humans, but only a small fraction have effective preventive vaccines or approved therapeutic agents [1, 2]. Moreover, the vast majority of viruses possess RNA-based genomes, which greatly increases their mutation rate and possibility for drug resistance, presenting considerable challenges for antiviral drug development. Beyond the acute phase, the global impact of COVID-19 continues to persist. Studies have demonstrated that residual viral RNA or antigens in some patients still give rise to a spectrum of persistent sequelae defined as "Long COVID" [3]. Timely antiviral treatment is anticipated to reduce the risk of developing Long COVID. The global challenges raised by the COVID-19 pandemic remind us that there is still a pressing need for novel antiviral strategies.

Soluble decoy receptors that competitively inhibit the interaction between viral envelope Spike proteins and host cell receptors constitute an effective antiviral strategy. Angiotensin-converting enzyme 2 (ACE2) has been reported as an essential receptor for severe acute respiratory syndrome coronavirus 2 (SARS-CoV-2) cellular entry [4]. Multiple studies have demonstrated that SARS-CoV-2 acquires enhanced ACE2-binding affinity through evolution to maximize transmissibility [4–7]. Therefore, ACE2-based derivatives and mimetics have attracted considerable interest as antiviral strategies against SARS-CoV-2. However, growing evidence from viral neutralization assays has demonstrated that the inhibitory efficacy of standalone decoy receptor strategies remains inherently incomplete and markedly dose-dependent, owing to the involvement of co-receptors and accessory proteins alongside the diversity of viral entry mechanisms [8].

Many small-molecule inhibitors act intracellularly to disrupt the viral life cycle by blocking viral genome replication and transcription. Various oligonucleotides, including small interfering RNAs (siRNAs) and antisense oligonucleotides (ASOs), exploit sequence specificity to precisely suppress viral gene function, yielding significant advances in hepatitis B virus (HBV) treatment [9, 10]. However, most small-molecule drugs are susceptible to enzymatic degradation and off-target effects, which narrow the therapeutic window and restrict their broader clinical application [10]. Peptide nucleic acid (PNA) is a synthetic nucleic acid analogue. It exhibits strong resistance to both nucleases and proteases owing to the N-(2-aminoethyl)glycine backbone [11]. They can form stable duplexes or triplexes with target RNA or DNA, thereby suppressing viral gene transcription and translation through steric hindrance [12, 13]. The potent inhibitory effects of PNA on human immunodeficiency virus (HIV) and HBV replication have been validated in cellular models [14–16].

Here, we report ACE2-PNA, a novel bifunctional therapeutic conjugate generated by covalent linkage of ACE2-Fc to PNA. ACE2-PNA possesses dual antiviral activity: it retains the virus-neutralizing activity of a decoy receptor, and simultaneously exploits the high affinity of the ACE2-Spike interaction to enable co-internalization with incompletely neutralized virions via endocytosis. Within the endolysosomal compartment, the ACE2 moiety is degraded by lysosomal acid hydrolases, while PNA, owing to its inherent protease resistance, remains intact and is subsequently released into the cytoplasm. Once in the cytoplasm, PNA binds to target sequences within the viral genome, thereby disrupting the viral life cycle.

We describe this conjugate-based approach as a novel Receptor-Drug Conjugate (RDC) strategy. This strategy confers dual antiviral efficacy, acting both extracellularly and intracellularly, to comprehensively target the virus across multiple stages of its life cycle. Importantly, RDC exploits viral infection itself as the delivery trigger, thereby substantially reducing the off-target toxicity of antiviral agents and offering the potential to extend the therapeutic window.

## Results

### In vitroscreening identifies D-(Arg)_8_-PNA_21_ targeting the conserved ORF1ab region as the lead candidate for conjugation

**Fig. 1:**
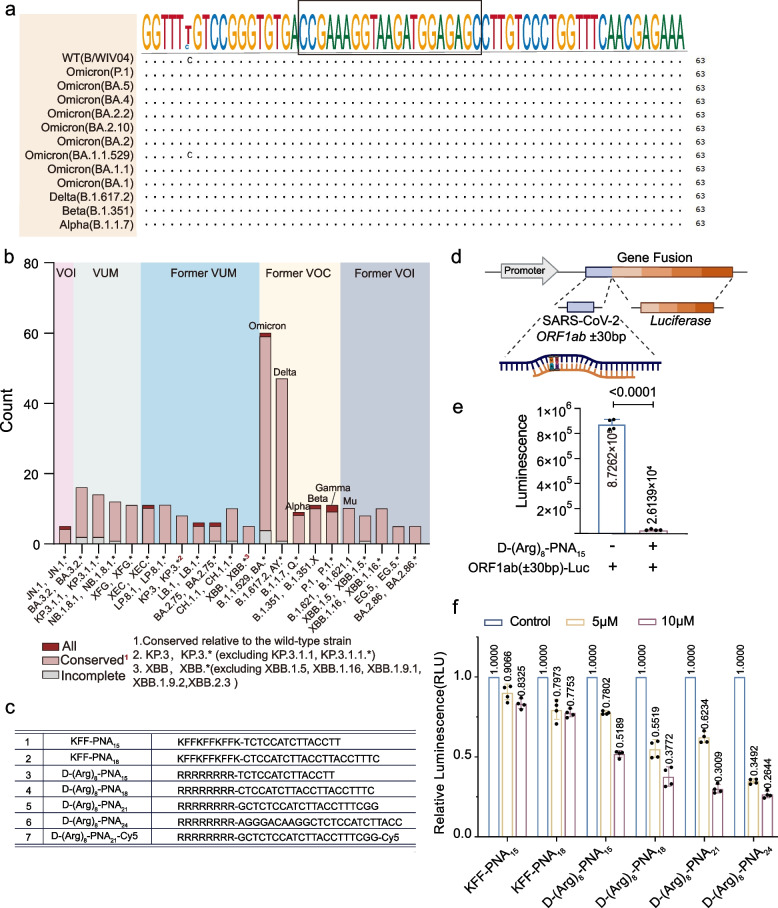
Design and Screening of CPP-PNAs. **a** Multiple sequence alignment of the *ORF1ab* ±30 bp region across SARS-CoV-2 sequences retrieved from the GISAID EpiCoV™ database, comprising 5 Variants of Concern (VOCs) and 8 Variants of Interest (VOIs) (data cutoff: October 9, 2022). Dots indicate conserved nucleotide positions. The black box denotes the D-(Arg)_8_-PNA_21_ target region. **b** Distribution of an expanded dataset comprising 287 high-coverage, complete SARS-CoV-2 sequences spanning all WHO-designated priority surveillance variant categories (data cutoff: April 20, 2026). For the two variants evaluated experimentally in this study, 47 sequences (Delta) and 60 sequences (Omicron) from distinct Pango lineages were independently sampled. For each category on the X-axis, the full height of the column represents the total number of sequences collected, the pink portion indicates the number of sequences with conserved *ORF1ab* ±30 bp identity relative to the wild-type strain, and the gray portion represents incomplete sequences excluded from multiple sequence alignment. **c** Sequences of all CPP-PNA conjugates employed in this study. PNA sequences are listed from 5′ to 3′.**d** Schematic of the reporter plasmid used for antisense activity screening of CPP-PNA candidates. CPP-PNA binds to the complementary target sequence within the SARS-CoV-2 *ORF1ab* ±30 bp region, sterically blocking translation and inhibiting luciferase expression. **e** CPP-PNA candidates were pre-mixed with the pcDNA3.1-*ORF1ab*(±30 bp)-*luciferase* reporter plasmid and co-transfected into HeLa cells. After 8 h, the medium was replaced with fresh medium and cells were cultured for a further 48 h. Luciferase activity was quantified by measuring fluorescence intensity (Y-axis) following substrate addition. D-(Arg)_8_-PNA_15_ is shown as a representative example. Data are mean ± SD (*n* = 4). Statistical significance was determined by an unpaired two-tailed Student's t-test. **f** CPP-PNA candidates at final concentrations of 5 or 10 μM were added to *ORF-Luc*-HeLa cells stably expressing the pcDNA3.1-*ORF1ab*(±30 bp)-*Luciferase*. After 8 h, the medium was replaced with fresh medium, and cells were cultured for a further 48 h. The Y-axis represents luciferase fluorescence intensity of each well normalized to the PBS-treated control group (shortened as *Control* in figure). The lower values indicate stronger antisense activity. Data are mean ± SD (*n* = 4)

As a positive-sense single-stranded RNA virus, SARS-CoV-2 firstly relies on host ribosomes to translate its *ORF1ab* reading frame upon cell entry. This process yields 16 non-structural proteins (NSP1-16) responsible for subsequent viral genome replication and transcription through proteolytic cleavage. Therefore, we sought to identify conserved sequences flanking the *ORF1ab* start codon as PNA target candidates, with the aim of enabling broad-spectrum inhibition across multiple SARS-CoV-2 variants. Sequences representing major circulating SARS-CoV-2 variants were retrieved from the GISAID EpiCoV™ database for multiple sequence alignment, comprising 5 Variants of Concern (VOCs) and 8 Variants of Interest (VOIs)(data cutoff: 9 October, 2022). The result revealed that the *ORF1ab* ±30 bp is highly conserved across all analyzed sequences and seen as a promising target for broad-spectrum PNA design (Fig. 1a). Given the ongoing emergence of novel SARS-CoV-2 variants, we expanded our sequence dataset to include 287 high-coverage, complete sequences spanning all WHO-designated priority surveillance variant categories (data cutoff: April 20, 2026), and further validated the conservation of the *ORF1ab* ±30 bp region across variants (Fig. 1b, Fig. S1, Table S1). Prior studies have established that the optimal length range for PNA is 15-25mer. Within this range, we designed a series of PNA sequences with lengths increasing in steps of 3 amino acids for further screening (Fig. 1c). All sequences spanned the *ORF1ab* start codon to enable efficient translational inhibition. In addition, cell-penetrating peptide (CPP) have been extensively reported to significantly enhance the intracellular delivery of PNA [16]. Among available CPPs, KFF or D-(Arg)_8_ were conjugated to candidate PNA.

The cellular uptake and antisense activity of CPP-PNA conjugates were assessed and compared using luciferase reporter assays (Fig. 1d). All six synthesized CPP-PNA conjugates were premixed with pcDNA3.1-*ORF1ab*(±30 bp)-*luciferase* plasmid prior to transfection into HeLa cells, and intracellular luciferase activity was reduced by more than 95% across all groups (data not shown). As a representative example, D-(Arg)_8_-PNA_15_ demonstrated robust antisense activity, suppressing luciferase expression by over 97% relative to the untreated control (Fig. 1e). Co-incubation of CPP-PNA conjugates with *ORF*-*Luc*-HeLa cells stably expressing the above reporter plasmid revealed that antisense activity increased progressively with PNA length across all six sequences. For a given PNA sequence, D-(Arg)_8_ seemed to provide superior cellular delivery and endosomal escape compared to KFF (Fig. 1f). However, when increased to 24 mer, D-(Arg)_8_-PNA_24_ exhibited significant cytotoxicity (Fig. S2a). BLASTn analysis against the human reference RNA sequence database(refseq_rna) revealed that, relative to D-(Arg)_8_-PNA_21_, D-(Arg)_8_-PNA_24_ exhibited a greater number of hits mapping to the 5′UTR and CDS regions of human gene transcripts, along with non-coding RNA (ncRNA) hits at lower E-values, indicating a greater risk of biologically meaningful off-target effects (Fig. S2b, Table S2). Furthermore, confocal microscopy of HeLa cells incubated with Cy5-labeled D-(Arg)_8_-PNA_21_ revealed persistent Cy5 fluorescence for more than 84 h (Fig. S2c), demonstrating prolonged intracellular stability of D-(Arg)_8_-PNA_21_. Based on these findings, D-(Arg)_8_-PNA_21_ was selected as the lead candidate for subsequent conjugation.

### ACE2-PNA retains the virus-neutralizing activity of ACE2-Fc

**Fig. 2:**
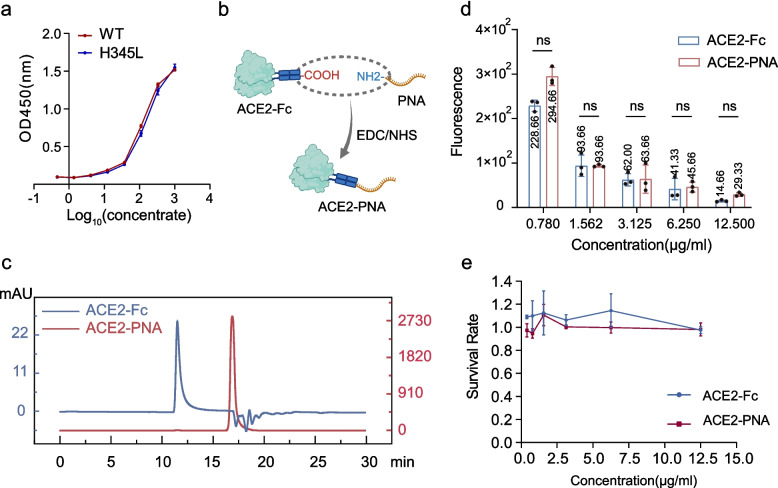
ACE2-PNA Retains the Virus-Neutralizing Activity of ACE2-Fc. **a** Binding affinity of wild-type ACE2-Fc and ACE2-Fc(H345L), shortened as *WT* and *H345L* respectively in figure, to the SARS-CoV-2 spike protein assessed by ELISA. 96-well plates were coated with SARS-CoV-2 spike protein (1 μg/mL), followed by incubation with serially diluted ACE2 proteins for 2 h. Bound protein was detected using an HRP-conjugated goat anti-human IgG (H + L) secondary antibody. Absorbance was measured at 450 nm. Data are mean ± SD (*n* = 3). **b** Schematic of EDC/NHS-mediated covalent conjugation of ACE2-Fc and CPP-PNA. EDC activates carboxyl groups on ACE2-Fc to form O-acylisourea intermediates, which react with NHS to generate stable NHS-esters. Upon adjustment to weakly alkaline pH (7–8), primary amines on CPP-PNA undergo nucleophilic attack to form stable amide bonds, yielding the ACE2-PNA conjugate. **c** RP-HPLC chromatogram of the ACE2-PNA reaction mixture following purification by ultrafiltration using a 50 kDa MWCO membrane. The ACE2-PNA conjugate displayed a dominant, symmetric peak at approximately 16.9 min, accounting for 98.99% of the total peak area. As a control, ACE2-Fc eluted at approximately 11.5 min. **d** Neutralization of SARS-CoV-2 spike pseudovirus by ACE2-Fc or ACE2-PNA. A fixed amount of SARS-CoV-2 spike-pseudovirus carrying a *luciferase* reporter gene was incubated with the indicated concentrations of ACE2-Fc or ACE2-PNA at 37 °C for 1 h, then added to *hACE2*-HEK293T cells. The medium was replaced after 8 h, and luciferase activity was measured 72 h post-infection. Higher fluorescence intensity indicates greater pseudovirus entry. Data are mean ± SD (*n* = 3). Statistical significance was determined by multiple unpaired Student's t-tests. Groups labeled "ns" indicate no statistically significant difference (P > 0.05). **e** CCK-8 assay of *hACE2*-HEK293T cells treated with the indicated concentrations of ACE2-Fc or ACE2-PNA. Following incubation, CCK-8 solution was added and absorbance was measured at 450 nm. Cell survival rate was calculated as the ratio of absorbance in each treatment group to that of the PBS-treated control. Data are mean ± SD (*n* = 3)

Multiple ACE2 mutation strategies aimed at abolishing peptidase activity for antiviral applications have been widely reported [7, 17–19]. Drawing on previous studies, we constructed the *ACE2*(H345L/1-740)-Fc plasmid, expressed it in 293 F cells, and purified via Protein A affinity chromatography (Fig. S3a). Enzymatic activity assays employing the fluorescent surrogate substrate Mca-APK(Dnp) and the physiological substrate Apelin-13 demonstrated complete abolishment of carboxypeptidase activity in ACE2-Fc(H345L) [20] (Fig. S3b-c). ELISA confirmed that wild-type ACE2-Fc and the H345L mutant exhibited comparable binding affinity to the SARS-CoV-2 Spike (S) protein (Fig. 2a). These results indicate that ACE2-Fc(H345L) retains Spike-binding capacity while losing carboxypeptidase activity, consistent with previous reports.

The ACE2-PNA conjugate was synthesized via 1-Ethyl-3-(3-dimethylaminopropyl)carbodiimide/N-Hydroxysuccinimide(EDC/NHS)-mediated amide bond formation (Fig. 2b). To assess conjugation efficiency and product purity, the conjugate was characterized by reverse-phase high-performance liquid chromatography (RP-HPLC) (Fig. 2c, Fig. S3d, Table S3). The reaction mixture was desalted by ultrafiltration through a 50 kDa molecular weight cut-off (MWCO), and removed unreacted PNA and cross-linking reagents. The chromatogram displayed a dominant, symmetric major peak, which was identified as the newly formed conjugate product based on its characteristic retention time. This peak accounted for 98.98% of the total chromatographic peak area, confirming that the conjugate was the predominant species in the post-reaction mixture and that purity was sufficient for subsequent in vitro and in vivo assays. Native mass spectrometry (nMS) of the ACE2-PNA conjugate yielded a broad charge-state envelope centered at approximately m/z 8000, consistent with previously report [21] (Fig. S3e). Owing to the difficulty in resolving individual charge states, the precise conjugation ratio between ACE2-Fc and CPP-PNA could not be determined.

The neutralizing activity of ACE2-PNA against SARS-CoV-2 was evaluated using a SARS-CoV-2 Spike pseudovirus carrying *luciferase* reporter gene and compared with ACE2-Fc. The results demonstrated that PNA conjugation did not affect the neutralizing potency of the ACE2 moiety (Fig. 2d). Furthermore, Cell Counting Kit-8 (CCK-8) assay results confirmed that neither ACE2-PNA nor ACE2-Fc exhibited appreciable cytotoxicity under the working concentration (Fig. 2e).

### ACE2-PNA demonstrates superior antiviral efficacy over ACE2-Fc in live virus assays

**Fig. 3:**
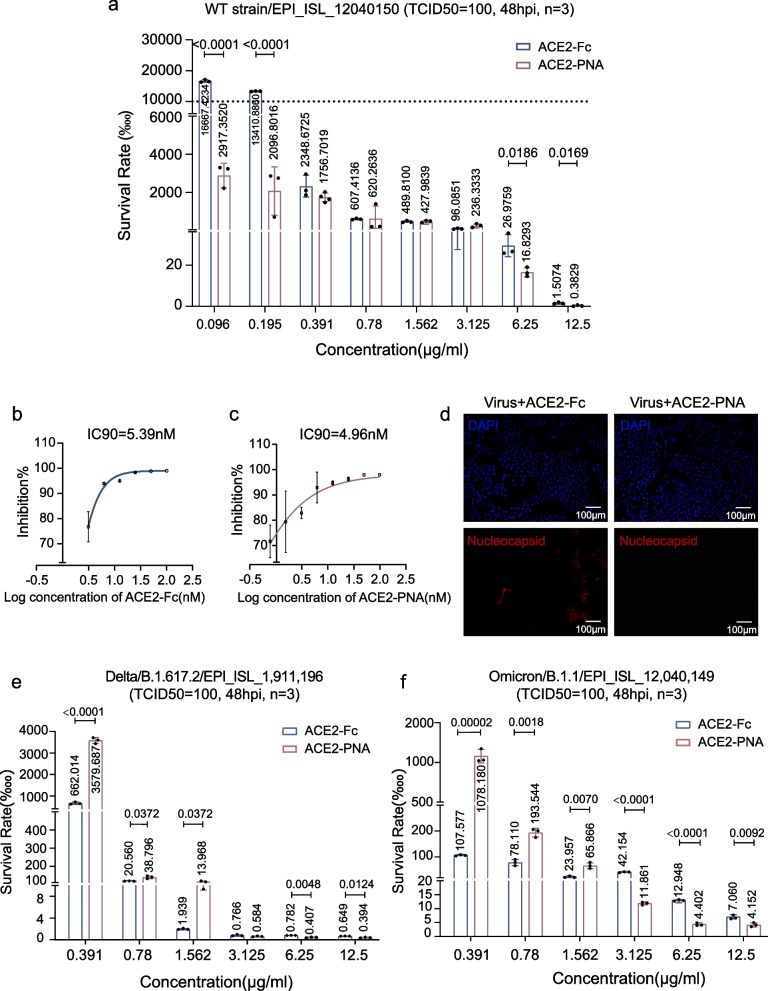
ACE2-PNA exhibits superior antiviral efficacy over ACE2-Fc in live virus experiments. **a** Inhibition of SARS-CoV-2 wild-type strain (EPI_ISL_12040150) infection in Vero E6 cells (TCID50 = 100) by ACE2-Fc or ACE2-PNA. **b-c** IC_90_ values of ACE2-Fc (**b**) and ACE2-PNA (**c**) against SARS-CoV-2 wild-type strain (EPI_ISL_12040150) infection in Vero E6 cells, derived from the dose–response data in (**a**) using GraphPad Prism 10. **d** Immunofluorescence images of Vero E6 cells infected with SARS-CoV-2 wild-type strain (EPI_ISL_12040150) in the presence of 6.25 μg/mL ACE2-Fc or ACE2-PNA. Intracellular viral particles were detected using anti-SARS-CoV-2 nucleoprotein rabbit monoclonal antibody. Cell nuclei were labeled with DAPI. Scale bar:100 μm. **e–f** Inhibition of SARS-CoV-2 Delta/B.1.617.2/EPI_ISL_1911196 (**e**) or Omicron/B.1.1/EPI_ISL_12040149 (**f**) infection in Vero E6 cells (TCID50 = 100) by ACE2-Fc or ACE2-PNA. For panels (**a**) (**e**) (**f**), the Y-axis represents relative viral survival rate normalized to the untreated control group. Data are mean ± SD (*n* = 3). Statistical significance was determined by multiple unpaired Student's *t*-tests. Only comparisons with *P* < 0.05 are indicated with connecting lines and values on the figure

To assess antiviral efficacy of ACE2-Fc and ACE2-PNA against the live SARS-CoV-2 wild-type strain (EPI_ISL_12040150) in Vero E6 cells, cell culture supernatant collected 24 h after removal of the viral inoculum were subjected to quantitative reverse transcription PCR (qRT-PCR) to quantify viral load. As shown in Fig. 3a, at 6.25 μg/mL, residual viral load in the ACE2-Fc group was 1.78-fold higher than in the ACE2-PNA group, corresponding to 3 × 10^-3^ and 1.68 × 10^-3^ of the untreated control, respectively. At 12.5 μg/mL, this difference was further amplified, with residual viral load in the ACE2-Fc group 3.94-fold higher than in the ACE2-PNA group(1.5074 × 10^-4^ vs. 0.3829 × 10^-4^ relative to the untreated control). The Ct value was subsequently converted to a reference value for absolute viral copy number using droplet digital PCR [22] (Fig. S4a). At 6.25 μg/mL, there remained 16.63 copies/mL in ACE2-Fc treated group, and 8.83 copies/mL in ACE2-PNA treated group; at 12.5 μg/mL, they were 0.64 copies/mL (ACE2-Fc) and 0.15 copies/mL (ACE2-PNA) (Fig. S4b). The IC_90_ values were determined to be 5.39 nM for ACE2-Fc and 4.96 nM for ACE2-PNA (Fig. 3b-c). In addition, ACE2-PNA consistently inhibited viral replication across all tested concentrations, while ACE2-Fc exhibited a paradoxical enhancement of viral replication at concentrations below 0.195 μg/mL. We hypothesize that this enhancement may arise from receptor-mediated endocytosis or antibody-dependent enhancement (ADE), and that PNA conjugation sterically abolishes this effect through structural occlusion.

Immunofluorescence results were consistent with these findings. At 6.25 μg/mL, residual fluorescence signal remained detectable in the ACE2-Fc group, whereas no signal was detected in the ACE2-PNA group (Fig. 3d, Fig. S5).

### ACE2-PNA exhibits pan-variant antiviral activity against SARS-CoV-2

The antiviral activity of ACE2-PNA against two globally prevalent SARS-CoV-2 variants, Delta/B.1.617.2/EPI_ISL_1,911,196 and Omicron/B.1.1/EPI_ISL_12,040,149, was further evaluated in Vero E6 cells. For the Delta variant, residual viral loads in the ACE2-Fc group were 1.9-fold (at 6.25 μg/mL) and 1.6-fold (at 12.5 μg/mL) higher than in the ACE2-PNA group (Fig. 3e, Fig. S4c). For the Omicron variant, residual viral loads in the ACE2-Fc group were 2.9-fold (at 6.25 μg/mL) and 1.7-fold (at 12.5 μg/mL) higher than in the ACE2-PNA group (Fig. 3f, Fig. S4d). That is to say, at concentrations ≥ 6.25 μg/mL, ACE2-PNA consistently demonstrated superior antiviral activity against all tested SARS-CoV-2 variants compared to ACE2-Fc. This pan-variant efficacy further corroborates the preservation of decoy receptor-mediated neutralization in ACE2-PNA and validates the rational design of the PNA target sequence.

### Endocytic viral entry constitutes a prerequisite for the intracellular delivery of ACE2-PNA

**Fig. 4:**
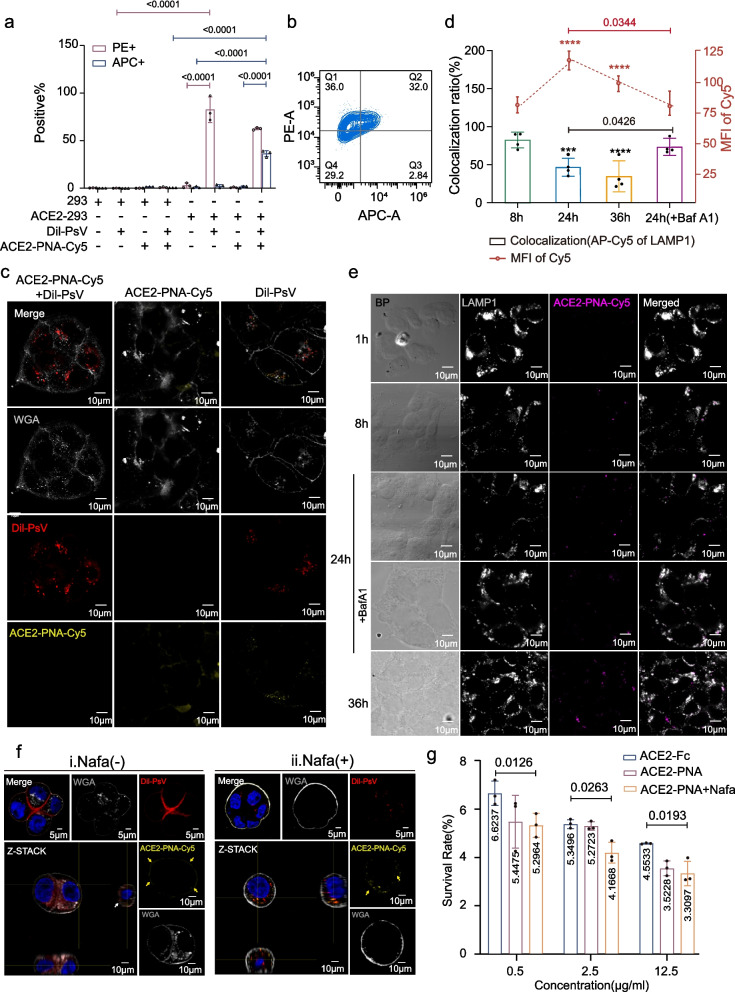
Endocytic viral entry constitutes a prerequisite for the intracellular delivery of ACE2-PNA. **a-b** HEK293 and *hACE2*-HEK293 cells were infected with Dil-labeled SARS-CoV-2 spike pseudovirus (shortened as *Dil-PsV* in Fig. 4), ACE2-PNA-Cy5, or a combination of both. The percentages of Dil-positive and Cy5-positive cell were quantified by flow cytometry (PE channel for Dil, APC channel for Cy5), with results summarized in (**a**), date are mean ± SD (*n* = 3). The representative flow cytometric dot plots in (**b**) show the cellular uptake of Dil-PsV and ACE2-PNA-Cy5 in hACE2-HEK293 cells infected by both. **c** Vero E6 cells were infected with Dil-labeled SARS-CoV-2 spike pseudovirus that pre-incubated with ACE2-PNA-Cy5 or not for 1 h. The viral inoculum was removed 4 h post-infection, and cells were fixed with 4% paraformaldehyde. Cell membranes were labeled with Alexa Fluor 488-WGA. Scale bar: 10 μm. **d** Mean fluorescence intensity of the Cy5 signal (MFI of Cy5, red dots with error bars; right Y-axis) and Manders' Colocalization Coefficients between Cy5 and LAMP1 signals (Colocalization ratio, bars with error bars; left Y-axis) at 8, 24, and 36 h post-infection, with or without 100 nM Bafilomycin A1 treatment. Quantification was performed using ImageJ. Asterisks indicate statistically significant differences between that time point and the 8-h group. P values for the Bafilomycin A1-treated group represent comparisons with the corresponding untreated group. Date are mean ± SD (*n* = 4) **e** Confocal microscopy images showing intracellular localization of ACE2-PNA-Cy5 at the indicated time points post-infection. Lysosomes were labeled with an anti-LAMP1 antibody. Scale bar: 10 μm. **f** Nafamostat (shortened as *Nafa* in figure)-pretreated Calu-3 cells were infected with Dil-labeled pseudovirus and ACE2-PNA-Cy5 and fixed for confocal imaging. Cell membranes and nuclei were labeled with Alexa Fluor 488-WGA and DAPI, respectively. Yellow arrows indicate the colocalization of ACE2-PNA-Cy5 and Dil-PsV. Z-stack images were acquired at step intervals of 0.2 μm. 3D reconstructions are shown in Supplementary Videos 1 and 2. **g** Inhibition of SARS-CoV-2 Delta variant/B.1.617.2/EPI_ISL_1911196 infection in Nafamostat-treated Calu-3 cells by ACE2-Fc or ACE2-PNA. Date are mean ± SD (*n* = 3). Statistical significance of (**a**) (**d**) was determined by one-way ANOVA, and of (**g**) was determined by two-way ANOVA

ACE2-PNA was designed to establish a virus infection-gated delivery system, in which intracellular drug delivery requires both viral presence and the infection of target cells. We used Cy5-labeled ACE2-PNA (ACE2-PNA-Cy5, Fig. S3d, Table S3) and Dil-labeled SARS-CoV-2 Spike pseudovirus (Dil-PsV) to validate this hypothesis. The Cy5 moiety was covalently conjugated to the C-terminus of PNA to ensure intracellular tracking of PNA localization. At 4 h post-infection, flow cytometric analysis revealed a significant increase in the Cy5-positive cell population exclusively in virus-infected cells (Fig. 4a-b, and Fig. S6a). In the absence of viral infection, no significant increase in the Cy5-positive population was detected, even upon treatment with ACE2-PNA-Cy5. Consistent results were observed in Vero E6 cells (Fig. 4c).

To investigate PNA cytoplasmic release, post-infection cellular imaging was performed using the lysosome-specific marker LAMP1 in conjunction with ACE2-PNA-Cy5 (Fig. 4d-e). The colocalization ratio between PNA and lysosomes was quantified at 8, 24, and 36 h post-infection and applied as a negative index of PNA cytoplasmic release. It was observed that, compared to 8 h post-infection, the colocalization ratio of Cy5 with LAMP1 progressively declined at the two later time points (24 and 36 h), while the intracellular mean fluorescence intensity (MFI) of Cy5 was significantly increased, indicating intracellular PNA accumulation. Bafilomycin A1 (100 nM), a inhibitor of endosomal acidification, significantly restored the colocalization ratio between Cy5 and LAMP1 at 24 h post-infection. Prolonged bafilomycin A1 treatment for 36 h resulted in widespread cell death, making the data impossible to analyse. Based on these findings, we proposed the following mechanistic model for PNA cytoplasmic delivery: following cellular internalization via endocytosis, the ACE2 moiety undergoes denaturation under acidic conditions and is subsequently degraded by lysosomal acid hydrolases. PNA, owing to its inherent protease resistance, remains and subsequently achieves lysosomal escape via cell-penetrating peptide-mediated or physically-driven membrane permeation, thereby entering the cytoplasm to exert its antiviral activity.

### Nafamostat restores ACE2-PNA antiviral activity in TMPRSS2-expressing cells by redirecting viral entry to the endocytic pathway

The entry route of SARS-CoV-2 is determined by the proteases responsible for Spike activation on the host cell, and the subcellular site of activation determined the location where viral membrane fusion occurs [23]. In addition to the endocytic pathway, SARS-CoV-2 typically exploits membrane-anchored TMPRSS2 to activate the spike in cells with high TMPRSS2 expression, enabling viral membrane fusion directly at the plasma membrane. That results in ACE2-PNA, which bound to the viral Spike, retaining at the cell surface. Consistent with this, imaging of Calu-3 cells (which highly express TMPRSS2) revealed colocalization of the Dil signal with the WGA-labeled plasma membrane signal, with ACE2-PNA-Cy5 similarly retained at the cell surface (Fig. 4f(i), Supplementary Video1). Prior studies have demonstrated that SARS-CoV-2 employs a flexible entry strategy, capable of shifting its preferred entry route in response to pharmacological pressure [23, 24]. Treatment with 1 nM Nafamostat resulted in the reappearance of intracellular Dil and Cy5 signals, similar to the pattern in Vero E6 cells (Fig. 4c), with colocalization between the two signals (Fig. 4f(ii); Supplementary Video2).

When Calu-3 cells were infected with the SARS-CoV-2 Delta (B.1.617.2) variant, which predominantly enters cells via plasma membrane fusion [25, 26], no significant difference in residual viral load was observed between the ACE2-Fc and ACE2-PNA treatment groups (Fig. 4g, Fig. S4e). Upon treatment with 1 nM Nafamostat, ACE2-PNA once again exhibited significantly superior antiviral efficacy relative to ACE2-Fc. Given that the Nafamostat concentration employed was below the threshold required to directly inhibit viral infection [27], we propose that Nafamostat-mediated TMPRSS2 inhibition redirected a large amount of viruses, which originally utilized the membrane fusion pathway, to the endocytic entry route. Under these conditions, ACE2-PNA can be co-internalized with the virus via endocytosis, enabling PNA to reach the cytoplasm and exert its antiviral activity, thereby restoring the full antiviral potency of ACE2-PNA.

### ACE2-PNA achieves virus-mediated targeted cellular entry in vivo

**Fig. 5:**
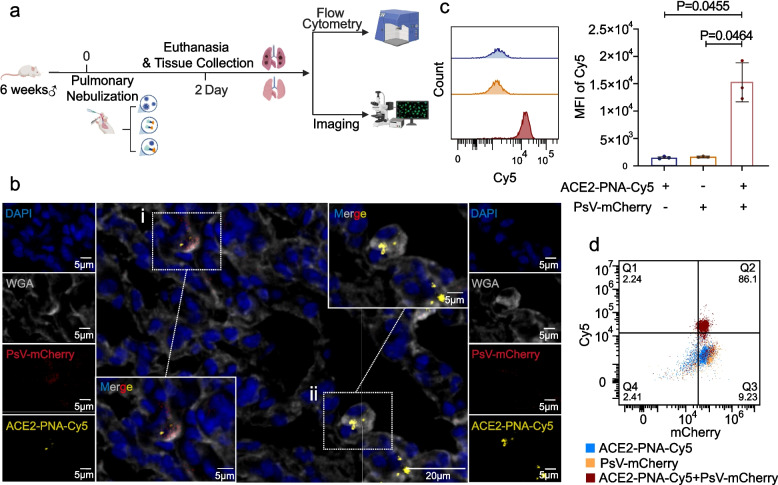
ACE2-PNA achieves virus-mediated targeted cellular entry in vivo*.* All procedures were carried out according to the timeline depicted in (**a**). 6-weeks male K18-*hACE2* mice were randomly divided into three groups to receive SARS-CoV-2 spike pseudovirus co-expressing mCherry and luciferase reporter genes (shortened as *PsV-mCherry*), ACE2-PNA-Cy5, or a combination of both via pulmonary nebulization. At 48 h post-administration, mice were euthanized and harvested lung tissues for further analysis. **b** Cryosection confocal images illustrating the colocalization of mCherry and Cy5 signals within cells (i) or outside (ii). Cell membranes and nuclei were labeled with Alexa Fluor 488-WGA and DAPI, respectively. Scale bar: 20 μm. **c** Histograms of Cy5 fluorescence intensity in single-cell suspensions from each group. Data are mean ± SD (*n* = 3). Statistical significance was determined by one-way ANOVA. **d** Representative flow cytometric scatter plot showing the distribution of lung single cells from the three treatment groups in the APC and PE channels (PE channel for mCherry, APC channel for Cy5)

To evaluate the in vivo delivery potential of ACE2-PNA, we administered SARS-CoV-2 Spike pseudovirus carrying *mCherry* and *luciferase* reporter genes, ACE2-PNA-Cy5, or a mixture of both, to C57BL/6JGpt-H11^em1Cin(K18−hACE2)^/Gpt mice (shortened as K18-*hACE2* mice) via pulmonary nebulization (Fig. 5a). Intracellular mCherry fluorescence was observed in lung tissue sections from pseudovirus-treated mice, confirming successful viral infection (Fig. S6b). Exclusively in lung tissue sections from the combination-treated group, we observed the intracellular Cy5 signal with notable colocalization with the mCherry signal (Fig. 5b). Flow cytometric analysis further confirmed that Cy5 fluorescence was detected only in lung cells from combination-treated mice, with nearly all Cy5-positive cells co-expressing mCherry (Fig. 5c-d, Fig. S6c). Furthermore, ACE2-PNA administration did not cause appreciable toxicity in mouse lungs (Fig. S6d-e). These results demonstrate that ACE2-PNA can be successfully co-internalized into target cells via virus-mediated endocytosis in vivo*,* and viral infection still represents a prerequisite for ACE2-PNA delivery in vivo, consistent with our in vitro observations.

## Discussion

**Fig. 6:**
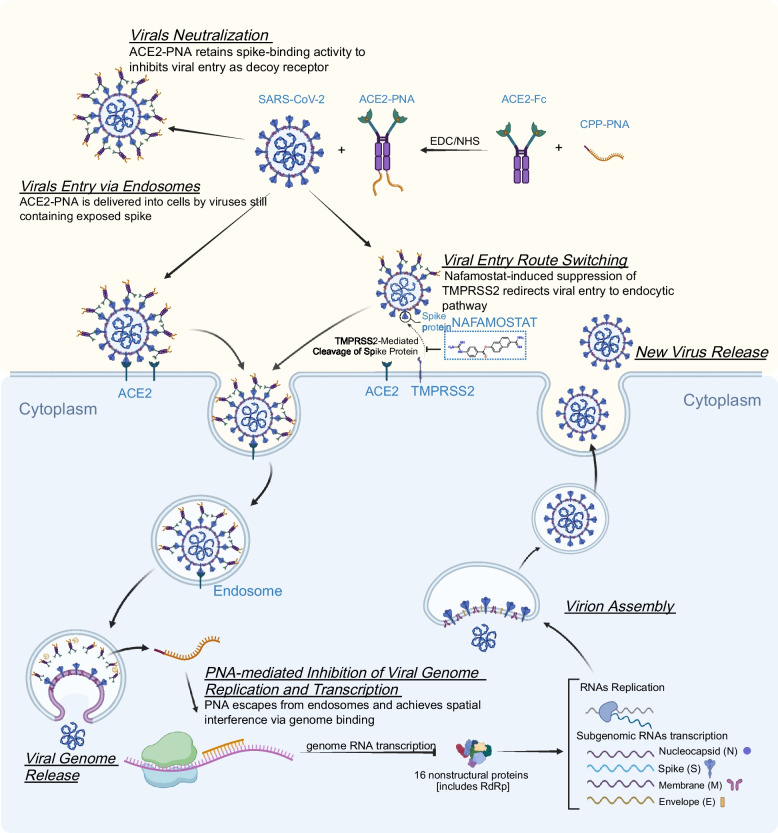
Working model of ACE2-PNA: A bifunctional antiviral strategy delivered via virus-mediated endocytosis. ACE2-PNA is a novel ACE2 derivative generated by covalent conjugation of PNA to ACE2-Fc, conferring dual antiviral efficacy acting both extracellularly and intracellularly. Here, Covalent conjugation was achieved via EDC/NHS-mediated amide bond formation. ACE2-PNA exerts dual antiviral activity: It retains the virus-neutralizing activity of a decoy receptor and simultaneously exploits the high affinity of the ACE2-Spike interaction to enable co-internalization with incompletely neutralized virions via endocytosis. Within the endolysosomal compartment, the ACE2 moiety is degraded by lysosomal acid hydrolases, while PNA, owing to its inherent protease resistance, remains intact and is subsequently released into the cytoplasm. Once in the cytoplasm, PNA binds to its target sequences (here designed within the SARS-CoV-2 *ORF1ab* ±30 bp region), thereby disrupting the viral life cycle. This figure is created in BioRender. Wang, Y. (2026) https://BioRender.com/79vo88s

We report a novel ACE2 derivative generated by covalent conjugation of PNA to ACE2-Fc to achieve dual antiviral efficacy acting both extracellularly and intracellularly. ACE2-PNA exploits the stable binding affinity between soluble ACE2 with the viral spike protein to co-internalize with incompletely neutralized virions into infected cells, where PNA continues to exert its antiviral activity (Fig. 6). This strategy overcomes the limitations of standalone decoy receptor strategies in the face of the virus' diverse and incompletely elucidated entry mechanisms.

Throughout evolution, SARS-CoV-2 has consistently maintained or acquired progressively enhanced ACE2-binding capacity to maximize transmissibility. The engineering of ACE2 has primarily focused on mutagenesis at the Spike-binding interface [28, 29], and fusion with an Fc domain to extend half-life in vivo. In addition, numerous engineered ACE2 variants and de novo-designed ACE2 mimetics have been reported. Among these, the engineered decoy receptor sACE2·v2.4 has been demonstrated to enhance RBD-binding affinity by over 35-fold relative to the wild-type [29].

In this study, we chose peptide nucleic acid (PNA) to construct the conjugate, owing to its high stability enabling sustained inhibition of viral genome replication. Moreover, PNA can be easily labeled with fluorescent group, facilitating investigation of the RDC cellular entry mechanism. The *ORF1ab* open reading frame encodes the first polyprotein to be translated following SARS-CoV-2 entry into host cells. PNA was designed to target the conserved sequence within this region, thereby achieving maximally efficient and broad-spectrum inhibition of the viral life cycle. A total of 300 high-coverage, complete SARS-CoV-2 sequences spanning all WHO-designated priority surveillance variant categories were retrieved from the GISAID EpiCoV™ database and subjected to multiple sequence alignment to assess the conservation of the D-(Arg)_8_-PNA_21_ target region in ongoing viral evolution (Fig. 1a-b, Fig. S1). The results validate the rational choice of the PNA target sequence and suggest that the antisense activity of D-(Arg)_8_-PNA_21_ is unlikely to be compromised by mutations arising in emerging viral variants. Antisense nucleotide strategies targeting other critical regions of the SARS-CoV-2 genome have also been reported, including the 5’ leader sequence and another highly conserved region, programmed ribosomal frameshifting stimulatory element (FSE), within *ORF1ab* [30, 31]. These regions represent promising targets for further optimization and screening of PNA sequences within the RDC framework.

Luciferase assay using SARS-CoV-2 Spike pseudovirus confirmed that ACE2-PNA retains virus-neutralizing activity comparable to that of ACE2-Fc. Live virus inhibition assays across three viral strains demonstrated that, at 12.5 μg/mL, the residual viral load in the ACE2-Fc group was at least 1.5-fold higher than in the ACE2-PNA group. We attribute this enhancement in antiviral efficacy to the intracellular activity of PNA. The concordance between relative viral load and absolute viral copy numbers obtained from parallel qRT-PCR and ddPCR. The values for viral RNA copy numbers was used only to provide approximate reference, the survival rate comparisons remain the primary basis for our conclusions.

The progressive increase of intracellular Cy5 MFI at later post-infection time points in cell-based experiments, and the concurrent decline in its colocalization coefficient with LAMP1, suggest that PNA undergoes gradual release from the lysosomal compartment over time. Pharmacological inhibition of endolysosomal acidification abolished this trend, further implicating the acidic endolysosomal microenvironment as an important role in PNA lysosomal release. Previous studies have demonstrated that the SARS-CoV-2 Spike protein binds to the Sorting Nexin 27 (SNX27) motif of ACE2, thereby inhibiting ACE2 endocytic recycling and directing its degradation within late endosomes/lysosomes [32]. On this basis, we propose the following mechanistic model for PNA release following ACE2-PNA cellular internalization: the ACE2 moiety undergoes denaturation under acidic conditions and is subsequently degraded by lysosomal acid hydrolases following cellular internalization via endocytosis, whereas PNA, owing to its inherent protease resistance, remains and subsequently achieves lysosomal escape via cell-penetrating peptide-mediated or physically-driven membrane permeation, thereby entering the cytoplasm to exert its antiviral activity. Nevertheless, the precise mechanism of PNA lysosomal escape remains to be further elucidated. Prospectively, cis-aconityl linkers is stable at physiological pH but subject to specific cleavage under acidic endolysosomal conditions. It can be incorporated into ACE2-PNA conjugates, to further facilitate conjugate dissociation and enhance intracellular PNA release efficiency [33, 34].

Under Nafamostat treatment, a substantial proportion of virions, which originally utilized the membrane fusion pathway, were redirected toward the endocytic pathway. Under these conditions, intracellular Cy5 signal was detected in ACE2-PNA-treated Calu-3 cells, confirming that ACE2-PNA was successfully co-internalized into target cells via virus-mediated endocytosis. Consistent with restored intracellular delivery, ACE2-PNA once again demonstrated significantly superior antiviral efficacy relative to ACE2-Fc in live virus assays.

Antibody-dependent enhancement (ADE) arises from the interaction between the Fc domain and Fc receptors on the cell surface, which facilitates viral infection [35]. The observed promotion of viral replication by ACE2-Fc at low concentrations in live virus assays may be attributable to this mechanism. However, ACE2-PNA did not exhibit this enhancement effect at low concentrations. We hypothesize that PNA conjugation alters the overall conformation of the complex, thereby sterically hindering the interaction of the ACE2 or Fc domain with their respective cellular receptors.

Although our study establishes proof of concept for a virus infection-activated bifunctional antiviral strategy, several limitations should be acknowledged. In this study, ACE2-Fc was conjugated to CPP-PNA via EDC/NHS-mediated chemistry. First, as each ACE2-Fc dimer contains multiple reactive carboxyl groups, the conjugation reaction potentially yields a heterogeneous product mixture. Furthermore, precise determination of the ACE2-to-PNA conjugation ratio failed to be achieved by native mass spectrometry (MS), owing to the complex glycosylation of ACE2. Second, live virus in vivo antiviral experiments were not performed. In future studies, we plan to conduct a comprehensive preclinical evaluation of ACE2-PNA based on achieving a homogeneous conjugate, including systematic live virus challenge experiments to validate its in vivo antiviral efficacy.

Our study introduces a virus-activated, dual-activity antiviral strategy, herein described as the Receptor-Drug Conjugate (RDC). While lipid nanoparticles (LNPs) represent a well-established and broadly adopted delivery platform, the RDC strategy uniquely confines drug release to virus-infected cells, substantially mitigating the off-target toxicity associated with nonspecific uptake of small-molecule drugs. Furthermore, the RDC strategy holds considerable potential for flexible adaptation to diverse viral pathogens. For example, substitution of ACE2 with intercellular adhesion molecule-1 (ICAM-1) or the coxsackievirus-adenovirus receptor (CAR) could extend the RDC platform suitable for rhinovirus and enterovirus infections [36, 37]. Incorporation of the Dendritic Cell-Specific Intercellular adhesion molecule-3-Grabbing Non-integrin (DC-SIGN) carbohydrate recognition domain (CRD) or the AXL receptor tyrosine kinase (AXL) domain could enable RDC to adapt to arboviral infections, including dengue and Zika virus disease [38, 39]. For viruses that depend on dual co-receptor engagement, such as HIV, a bispecific chimeric RDC construct incorporating dual decoy receptor moieties could be further developed [40].

## Materials and methods

Key experimental procedures and materials are summarized herein; more detailed protocols are available in the Supplementary Material.

### Cells and virus

The Calu-3 cells were purchased from Procell Life Science & Technology Co., Ltd. (Wuhan, China; catalog no.CL-0054). The Vero E6 and *hACE2*-HEK293T cells were kindly provided by the Zhejiang Provincial Center for Disease Control and Prevention (Hangzhou, China). Calu-3 cells were cultured in RPMI-1640 medium (Gibco, USA, C11875500BT) supplemented with 20% fetal bovine serum (FBS; Gibco, USA, A5256701) and 1 × nonessential amino acids (Sigma, USA, 11,140,050). All other cells were cultured in Dulbecco’s modified Eagle’s medium (DMEM; Gibco, USA, C11995500BT) supplemented with 10% fetal bovine serum. All these cells were cultured at 37 °C with 5% CO_2_ and routinely tested for mycoplasma contamination.

The *ORF-Luc*-HeLa cell line, which stably expresses ORF1ab(±30 bp)-Luciferase, was generated by transfection with the pcDNA3.1-*ORF1ab* ±30 bp-*Luciferase* plasmid followed by antibiotic selection with G418. Selection was performed at 700 μg/mL G418 for three weeks, after which the concentration was reduced to 350 μg/mL for routine maintenance.

SARS-CoV-2 Spike pseudoviruses were purchased from VectorBuilder (Guangzhou, China; P240716-1011urc, P250417-1358urz). The following live SARS-CoV-2 strains were obtained from the Zhejiang Provincial Center for Disease Control and Prevention (Hangzhou, China): SARS-CoV-2/Vero/WGF/2020/WZ122 (wild-type; GISAID: EPI_ISL_12040150), SARS-CoV-2/Vero/LXG/2021/ZJ28 (Delta, B.1.617.2; GISAID: EPI_ISL_1911196), and SARS-CoV-2/VeroE6/DSh/2021/ZJ25 (Omicron/B.1.1; GISAID: EPI_ISL_12040149). All strains were originally isolated from throat swabs and propagated in Vero cells. Viral titers were determined by microdose cytopathic effect (CPE) assay. Virus stocks were harvested upon observation of 50% CPE.

### PNA design and synthesis

All PNAs were synthesized and HPLC-purified by PANAGENE Inc. (Daejeon, Korea). 25 μmol powder was dissolved in ddH₂O to obtain a 200 μM stock solution, stored at -20 °C.

### Measurement of PNA antisense activity

PNA antisense activity was measured in a white 96-well plate. *ORF-Luc*-HeLa cells were detached and resuspended in Opti-MEM (Gibco, USA, 31,985,070) at 1 × 10^5^ cells/mL. CPP-PNA (5 or 10 μM) and chloroquine (CQ; 100 μM) were prepared in Opti-MEM. Each well received 100 μL of cell suspension (1 × 10^4^ cells) mixed with the CPP-PNA and CQ dilutions to a final volume of 200 μL. FBS (10% final concentration) was added 8 h post-transduction. After a further 48 h, intracellular luciferase activity was quantified using a One-Lumi™ Firefly Luciferase Reporter Gene Assay Kit (Beyotime, China, RG056S) and luminescence was measured on a BioTek Synergy H1 Multimode microplate reader.

### Conjugation of ACE2-PNA via the EDC/NHS reaction

The ACE2-Fc protein solution was diluted with 2-Morpholinoethanesulphonic acid (MES) buffer (0.1 M MES, 0.5 M NaCl, pH5.5). 1-Ethyl-3-(3-dimethylaminopropyl)carbodiimide (EDC; Aladdin, China, A638729; 1 mM final) and N-Hydroxysuccinimide (NHS; Aladdin, China, N164052; 5 mM final) were added sequentially with vortexing. The mixture was incubated at room temperature for 2 h. Subsequently, CPP-PNA was added at a molar ratio of 5:1 (PNA:ACE2-Fc), the pH was adjusted to 7.0-8.0 with 0.2 M sodium phosphate buffer, and the reaction was proceeded at 4 °C. Following overnight incubation, unreacted small-molecule reagents and PNA were removed by ultrafiltration using a 50 kDa MWCO centrifugal filter (Millipore, Germany, UFC5050) and the buffer was exchanged to phosphate-buffered saline (PBS). The conjugate was characterized by RP-HPLC using a Waters Protein-Pak™ Diol(OH) column (10 μm, 7.8 × 300 mm; Waters Corporation, USA; WAT085250).

### Neutralizing capacity assay using luciferase-pseudoviruses

The neutralizing activity of ACE2-Fc and ACE2-PNA was evaluated using a SARS-CoV-2 Spike pseudovirus carrying luciferase reporter gene (Guangzhou, China; P240716-1011urc). *hACE2*-HEK293T cells (1.5 × 10^4^ cells/well) were seeded in a white 96-well plate 1 day prior to infection to ensure 70%-80% confluence at the time of infection. ACE2-Fc or ACE2-PNA was two-fold serially diluted from 0.78 to 12.5 µg/mL in complete DMEM and pre-incubated with SARS-CoV-2 spike pseudoviruses at 37 °C for 1 h. The mixtures were then added to hACE2-HEK293T cells. After 48-72 h, luciferase activity was quantified using a One-Lumi™ Firefly Luciferase Reporter Gene Assay Kit (Beyotime, China, RG056S) and luminescence was measured on a BioTek Synergy H1 Multimode microplate reader.

### The internalization of Dil-Pseudovirus and ACE2-PNA-Cy5

#### Viral entry pathway analysis

Dil labeling of the pseudoviruses envelope was performed according to the manufacturer's instructions (Beyotime, China, C1036). One day prior to infection, cells were seeded in 48-well plates at 2.5 × 10^4^ (for Vero E6) or 4 × 10^4^ (for Calu-3 and *hACE2*-HEK293T) cells/well. Before the experiments were performed, the labeled virus was filtered through a 0.2 μm filter to remove aggregates and then pre-incubated with ACE2-PNA-Cy5 at 37 °C for 1 h. Cells were incubated with the mixture at 4 °C for 30 min, then transferred to 37 °C for a further 6 h. The medium was removed, and cells were washed three times with HHBS.

For confocal imaging, cells were fixed with 4% paraformaldehyde for 10 min, and stained with Alexa Fluor 488-WGA (Maokang Biotechnology, Chian, MP6329-1MG) and DAPI (Beyotime, China, C1002). Images were acquired on an Olympus FV3000 confocal laser scanning microscope (Olympus Corporation, Japan).

For flow cytometric analysis, cells were detached, stained with Zombie Violet™ Fixable Viability Kit (BioLegend, USA, 423,113), and acquired on a CytoFLEX S flow cytometer (Beckman Coulter, CA, USA).

Where indicated, cells were pre-treated with Nafamostat (MedChemExpress, USA, HY-B0190A;1 nM) for 1 h prior to the addition of the pseudovirus-conjugate mixture. Nafamostat was maintained in the culture medium throughout the experiment.

#### Lysosomal colocalization analysis

CM-Dil labeling of the pseudoviruses envelope was performed according to the manufacturer's instructions (Thermo Fisher Scientific, USA, C7000). Cells seeded and infected in parallel were fixed at 1, 8, 24, and 36 h post-infection, permeabilized with 0.1% digitonin(Yeasen, China, 54004ES25), and blocked with 3% BSA. Lysosomes were labeled with an anti-LAMP1 rabbit monoclonal antibody (D2D11; CST, USA, 9091; 1:300) incubated overnight at 4 °C, followed by DyLight 594-conjugated goat anti-rabbit IgG (Abbkine, China, A23420; 1:200) for 1 h at room temperature. Mean fluorescence intensity (MFI) and Manders' Colocalization Coefficients (MCC) were calculated using ImageJ (NIH, MD, USA).

Where indicated, cells were pre-treated with Bafilomycin A1 (MedChemExpress, USA, HY-100558; 100 nM) for 30 min at 4 °C followed by 30 min at 37 °C prior to addition of the pseudovirus-conjugate mixture. Bafilomycin A1 (100 nM) was maintained in the culture medium throughout the experiment.

### Live virus infection assay in the BSL-3 laboratory

All experiments involving live SARS-CoV-2 were conducted in the Biosafety Level 3 (BSL-3) laboratory at the Zhejiang Provincial Center for Disease Control and Prevention (Hangzhou, China). SARS-CoV-2 was pre-incubated with two-fold serially diluted ACE2-Fc or ACE2-PNA (0.78-12.5 μg/mL) at 37 °C for 2 h, after which the mixtures were used to infect cells at a titer of TCID50 = 100. 24 h post infection, the medium was replaced with complete medium supplemented with the corresponding concentrations of ACE2-Fc or ACE2-PNA for a further 24 h. Culture supernatants were collected for RNA extraction and qRT-PCR analysis, and cells were fixed with 4% paraformaldehyde for immunofluorescence staining.

Where indicated, cells were pre-treated with Nafamostat (MedChemExpress, USA, HY-B0190A; 1 nM) for 1 h prior to the addition of the virus-conjugate mixture. Nafamostat was maintained in the culture medium throughout the experiment.

### In vivopseudovirus infection model using mcherry/luciferase-pseudovirus and ACE2-PNA-Cy5

#### Animal procedures

6 weeks male C57BL/6JGpt-H11^em1Cin(K18−hACE2)^/Gpt mice (shortened as K18-*hACE2* mice), in which the human ACE2 protein is expressed under the control of the K18 promoter, were purchased from GemPharmatech (Nanjing, China, Certificate No. B202505230409). As pulmonary nebulization requires specialized technical expertise, all animal experiments were conducted in collaboration with a qualified contract research organization (CRO), Hangzhou HIBIO Technology Co., Ltd. (Hangzhou, China; China National Accreditation Service for Conformity Assessment laboratory accreditation No. LA0040; animal use license No. SYXK2023-0020), to ensure compliance with animal welfare standards. All procedures involving animal models were performed in compliance with ethical standards and were approved by the Institutional ethics committee (Approval No. HB2412025). Mice were maintained under specific pathogen-free conditions and provided a standard diet. Body weight and general behavior were monitored daily throughout the experimental period. After 48 h the mice were euthanised, and collected lung tissues for flow cytometry analysis or sectioning.

#### Preparation and staining of mouse lung cryosections

Lung tissues were excised and embedded in Tissue-Tek O.C.T. Compound (Sakura Finetek, Japan, 4583) for cryosectioning. Sections were stained with Alexa Fluor 488-WGA (Maokang Biotechnology, China, MP6329-1MG) to label cell membranes, and DAPI (Beyotime, China, C1002) to visualize nuclei. Fluorescence images were acquired on an Olympus FV3000 confocal laser scanning microscope (Olympus Corporation, Tokyo, Japan). Cryosectioning and staining were performed by Hangzhou Hulk Bio-Tech Co., Ltd. (Hangzhou, China).

#### Preparation of single-cell suspensions from mouse lung tissue

Approximately 0.5 mg tissue was excised and minced into small fragments with a scalpel, after which 1 mL of digestion solution (2 mg/mL Collagenase IV, Gibco, USA, 17,104,019; 25 μg/mL DNase I, Takara, Japan, 2270 A; 1 U/mL Dispase II, Yeasen, China, 40104ES60; 2% FBS, Gibco, USA, A5256701) was added. After mixing, the tissue was transferred to a 15 mL centrifuge tube, 4 mL of digestion solution was added, and the tissue was digested on a shaker at 60–80 rpm at 37 °C for 30-60 min. The digested tissue was filtered through a 70 μm cell strainer and collected into a centrifuge tube containing 5 mL of complete RPMI-1640 medium, an appropriate volume of red blood cell lysis buffer (BD, USA, 555,899) was added. The sample was incubated on ice for 5 min; then terminated by adding a 2 × volume of complete RPMI-1640 medium. After centrifuged at 450 × g for 10 min at 4 °C, the supernatant was discarded, and the pellet was resuspended in FACS buffer (2% FBS in RPMI-1640 medium) to obtain a single-cell suspension for flow cytometric analysis.

### Statistical analysis

All experiments were performed at least three times. The data are presented as the mean ± standard deviation (SD) values. Statistical significance for Fig. 1e was determined by an unpaired two-tailed Student's t-test; for Fig. 2d/3a/3e/3f was assessed using a multiple unpaired Student's t-tests; for Fig. 4g was analyzed using a two-way ANOVA; and all flow cytometry data was assessed using a one-way ANOVA. The analysis method applied for each dataset is indicated in the corresponding figure legends. Unless otherwise stated, only those with P value less than or equal to 0.05 was display values and lines on the figure. All analyses were performed using GraphPad Prism 10.

## Supplementary Information

Supplementary Material 1: Supplemental Methods and Figures.

Supplementary Material 2: Supplementary Table1: SARS-CoV-2 Sequences Used for Multiple Sequence Alignment.

Supplementary Material 3: Supplementary Table2: Blast results for PNA21 and PNA24 against the human reference RNA sequence database (refseq_rna).

Supplementary Material 4: Supplementary Table3: RP-HPLC data mentioned in this article.

Supplementary Material 5: Supplementary Video1: 3D reconstructions of the Z-stack images in Fig. 4F(i).

Supplementary Material 6: Supplementary Video2: 3D reconstructions of the Z-stack images in Fig. 4F(ii).

## Data Availability

All data needed to evaluate the conclusions in the paper are present in the paper and/or the Supplementary Materials. Further information and requests for resources and reagents should be directed to and will be fulfilled by the lead contact, CGZ (cgzhu@zju.edu.cn).
