# Supplementary material for: ACE2-PNA conjugates exploit viral endocytosis for targeted intracellular delivery and exhibit dual antiviral efficacy against SARS-CoV-2
Source: Mol Biomed. 2026 Aug 3;7:122. doi: 10.1186/s43556-026-00520-6 (PMC13433682; doi:10.1186/s43556-026-00520-6)
Supplement: Supplementary file 1 [file 43556_2026_520_MOESM1_ESM.pdf]

ACE2-PNA Conjugates Exploit Viral Endocytosis for Targeted Intracellular Delivery and Exhibit Dual Antiviral Efficacy Against SARS-CoV-2

Yifei Wang[1 †], Jinghan Xu[2 †], Yiquan Chen[1], Xin Xiao[1], Yiru Zhu[1], Liyang Yu[1], Wen Shi[2], Jingchao Li[1,3], Jianhua Li[2] *, Chenggang Zhu[1]*

[1] College of Life Sciences, Zhejiang University, Hangzhou, China

[2] Zhejiang Key Laboratory of Public Health Detection and Pathogenesis Research Department of Microbiology, Zhejiang Provincial Center for Disease Control and Prevention, Hangzhou, China

[3] School of Medical Technology and Information Engineering, Zhejiang Chinese Medical University, Hangzhou, China

*Corresponding author:Jianhua Li(jhli@cdc.zj.cn) , Chenggang Zhu(cgzhu@zju.edu.cn)

†These authors contributed equally to this work.

This Supplementary file contain:

- Supplemental Methods
- Supplemental Figures 1-6 and their associated legends

Supplemental Methods

Plasmid construction, purchase, and protein expression

The *ORF1ab*±30 bp sequence was obtained from NCBI (NC_045512.2). The pcDNA3.1(+) vector and luciferase sequence were derived from laboratory stocks. pcDNA3.1-ACE2(WT/1-740)-Fc-Myc-His was synthesized by Sunya Biotechnology Co., Ltd(Zhejiang, China), and the H345L mutation was introduced by PCR-based site-directed mutagenesis. Briefly, each reaction was carried out in a 0.2 mL tube in a total volume of 25 μL, containing 12.5 μL PrimeSTAR Max DNA Polymerase (Takara, Japan, R045A), 0.5 μL template DNA (≤10 ng), 0.5 μL of 10 μM forward primer, 0.5 μL of 10 μM reverse primer, and sterile deionized water to a final volume of 25 μL. PCR was performed using the following cycling conditions: 98°C for 5 min; 29 cycles of 98°C for 10 s, 55°C for 15 s, and 72°C for 40 s; followed by a final hold at 4°C. The amplification reaction was treated with 1 μL of the FastDigest DpnI restriction enzymes (Thermo Fisher Scientific, USA, FD1703) at 37°C for 30 min. All DpnI-treated products were transformed into E. coli DH5α competent cells. Single colonies were picked and cultured overnight for plasmid isolation. All plasmids were verified by Sanger sequencing to confirm the presence of the intended mutations and the absence of unintended sequence alterations. The ACE2(H345L)-Fc protein was secreted by HEK293F cells, purified using Protein A affinity resin, and produced by Nanjing Detai Bioengineering Co .,Ltd(Nanjing,China).

PNA design and synthesis

Multiple sequence alignment

Sequences of major circulating SARS-CoV-2 variants were retrieved from the GISAID EpiCoV™ database for the first alignment, comprising 5 Variants of Concern (VOCs) and 8 Variants of Interest (VOIs) (data cutoff: October 9, 2022).Given the continued emergence of novel SARS-CoV-2 variants, a random sample of 278 SARS-CoV-2 sequences was subsequently retrieved from the GISAID EpiCoV™ database for alignment to further substantiate the conservation of the ORF1ab ±30 bp region(data cutoff: April 20, 2026). Specifically, within each WHO-defined priority variant category (including all VOC, VOI,

VUM, Former VUM, Former VOC, and Former VOI), a minimum of 5 high-coverage, complete genome sequences from distinct Pango lineages were randomly selected, to construct a broadly representative alignment dataset spanning all priority surveillance variant categories. Sequence alignment was performed using SnapGene 6.0.2(Dotmatics, USA).

Off-Target Risk Analysis

We performed BLASTn searchs of PNA_{21} (5′-GCTCTCCATCTTACCTTTCGG-3′) and PNA_{24}(AGGGACAAGGCTCTCCATCTTACC) against the NCBI human reference RNA sequence database (refseq_rna) to evaluate potential off-target interactions. The search was conducted with the following parameters: database = refseq_rna; organism = *Homo sapiens* (taxid: 9606); optimized for short input sequences (Optimize for: Somewhat similar sequences; Word size = 7). In general, off-target effects are typically minimized by avoiding continuous complementarity with unexpected transcripts (typically >10-15 nucleotides). The melting temperature (Tm) of a 13-mer PNA-DNA hybrid has been reported to exceed 52°C, which is sufficient to mediate effective steric blocking within cells. Therefore, hits with query coverage >62% and percent identity of 100%,which implying ≥13-nt perfect complementarity, were selected for further analysis to access the off-target risks

ACE2–Spike binding assay

ACE2-spike binding affinity was evaluated by ELISA as previously described[20] with minor modifications. 96-well plates were coated with 100 μL of 1μg/mL SARS-CoV-2 Spike protein (S1; GenScript, China, Z03501) and incubated overnight at 4°C. After blocking for 1 h with 3% FBS at 37°C, serial dilutions of ACE2-Fc wild-type or mutant were added and incubated for 2 h at room temperature. After 3 washes with PBST, HRP-conjugated goat anti-human IgG (H+L) (Abmart, China, M22001M; 1:1000 dilution) was added as the secondary antibody. After a further 3 washes, 3,3',5,5'-tetramethylbenzidine dihydrochloride (TMB) was added as the substrate solution to the plates, and OD450 value was determined using a BioTek Synergy H1 Multimode Reader.

ACE2-Fc enzymatic assay

ACE2 carboxypeptidase activity was assessed as previously described[20] with minor modifications. For the fluorescent surrogate substrate assay, Mca-APK(Dnp) (Cayman Chemical, USA, 502103-1) was incubated with serial concentrations of wild-type or H345L mutant ACE2-Fc in reaction buffer (50 mM 4-morpholineethanesulfonic acid (MES), pH 6.5; 300 mM NaCl; 10 μM $ZnCl_2$; 0.01% Triton X-100) in a black 96-well plate at room temperature for 20min. For the physiological substrate assay, apelin-13 (MedChemExpress, USA, HY-P1944) was incubated with ACE2-Fc (wild-type or H345L mutant) in reaction buffer (20 mM Tris-HCl, pH 7.4; 136 mM NaCl; 10 μM $ZnCl_2$) at 37°C for 20 min. The reaction was terminated by heat inactivation at 80°C for 5 min. Released phenylalanine was subsequently quantified using a phenylalanine detection kit (Sigma, USA, MAK484) per the manufacturer's instructions.

Cytotoxicity Assay using a Cell Counting Kit-8

Cytotoxicity of CPP-PNAs was assessed in HeLa cells seeded at 1×10^4 cells per well. Cells were treated with each of the six CPP-PNA sequences at final aconcentration of 5 μM or 10 μM for 48 h. Then, 10 μL of CCK-8 solution (MedChemExpress, USA, HY-K0301) was added to each well, and the absorbance value at 450 nm was measured after 1 h using a BioTek Synergy H1 Multimode Reader.

For ACE2-Fc and ACE2-PNA, the cytotoxicity was evaluated in *hACE2*-HEK293 cells seeded at 1×10^4 cells per well. Cells were treated with two-fold serial dilutions of ACE2-Fc or ACE2-PNA (0.78-12.5μg/mL final) in complete DMEM for 48 h. Following treatment, cell viability was assessed using the CCK-8 assay as described above.

Procedures Following Live Virus Infection

The live virus infection procedure is described in the Materials and Methods section of the main text. The following section details the downstream experimental procedures conducted after infection.

Viral RNA extraction and qRT-PCR

Viral RNA in the cell culture supernatant was extracted using an RNeasy Mini Kit(QIAGEN, Germany, 74104), and the viral RNA load in a sample from each well was subsequently measured using a 2019 Novel Coronavirus (2019-nCoV) RNA detection kit (PCR-Fluorescence Probing, Daan Gene, China, DA-TF/nCoV-100 V2/0). qRT-PCR was performed in a LightCycler 480 instrument (Roche, Switzerland). Thermal cycling conditions were as follows: 50°C for 15 min (reverse transcription); 95°C for 15 min (initial denaturation); 45 cycles of 94°C for 15 s and 55°C for 45 s; followed by a final cooling step at 40°C for 30 s. Data were analyzed using GraphPad Prism software (GraphPad Software, San Diego, CA, USA).The data were analyzed using Prism software (GraphPad Software, CA, USA). The inhibition rate in each sample was calculated as follows: Inhibition%= (1-2 △△Ct) × 100%.

Droplet digital PCR

Droplet digital PCR was assessed as previously described[22]. Primers and probes targeting the SARS-CoV-2 nucleocapsid (N) and spike (S) genes were synthesized by Sangon Biotech (Shanghai, China). Droplet digital PCR (ddPCR) was performed using the One-Step RT-ddPCR Advanced Kit for Probes (Bio-Rad, USA, 1864021) according to the manufacturer's instructions. Thermal cycling conditions were as follows: reverse transcription at 45°C for 60 min; Taq polymerase activation at 95°C for 10 min; 40 cycles of denaturation at 95°C for 30 s and touchdown annealing at 60°C for 1 min; enzyme inactivation at 98°C for 10 min; and a final hold at 4°C. The following instruments were used: QX200™ Droplet Generator (Bio-Rad, USA), QX200 Droplet Reader (Bio-Rad, USA), Automated Droplet Generator (Bio-Rad, USA, 1864101), PX1 PCR Plate Sealer (Bio-Rad, USA,), and QIAcube HT Automated Nucleic Acid Purification System (QIAGEN, Hilden, Germany).The primer-probes used in this study:

For N gene:

Forward primer:ACATTGGCACCCGCAATCC

Reverse primer:GCTTGACTGCCGCCTCTGCT

Probe:FAM-5'-CGTGCTACAACTTCCTCAAGGAACA-3'-BHQ1

For S gene:

Forward primer:TTGATCACAGGCAGACTTCAAAGT

Reverse primer:AGCTCTGATTTCTGCAGCTCTAATT

Probe:VIC-5'-TGCAGACATATGTGACTCA-3'-BHQ1

Immunocytochemistry

After fixed with 4% paraformaldehyde , the cells were permeabilized with 0.01%Triton X-100, blocked with 3% BSA for 1h and incubated with an anti-SARS-CoV-2 Nucleoprotein rabbit monoclonal antibody (Abcam, UK, AB271180; 1:1000) at 4°C overnight. Alexa Fluor488®-conjugated goat anti-rabbit IgG (Abcam, UK, AB150077; 1:1500) was used as the secondary antibody at room temperature for 2h. Nuclei were stained with DAPI (Beyotime, China, C1002). Images were acquired on an Olympus FV3000 confocal laser scanning microscope (Olympus Corporation, Japan).

- **Supplemental Figures**

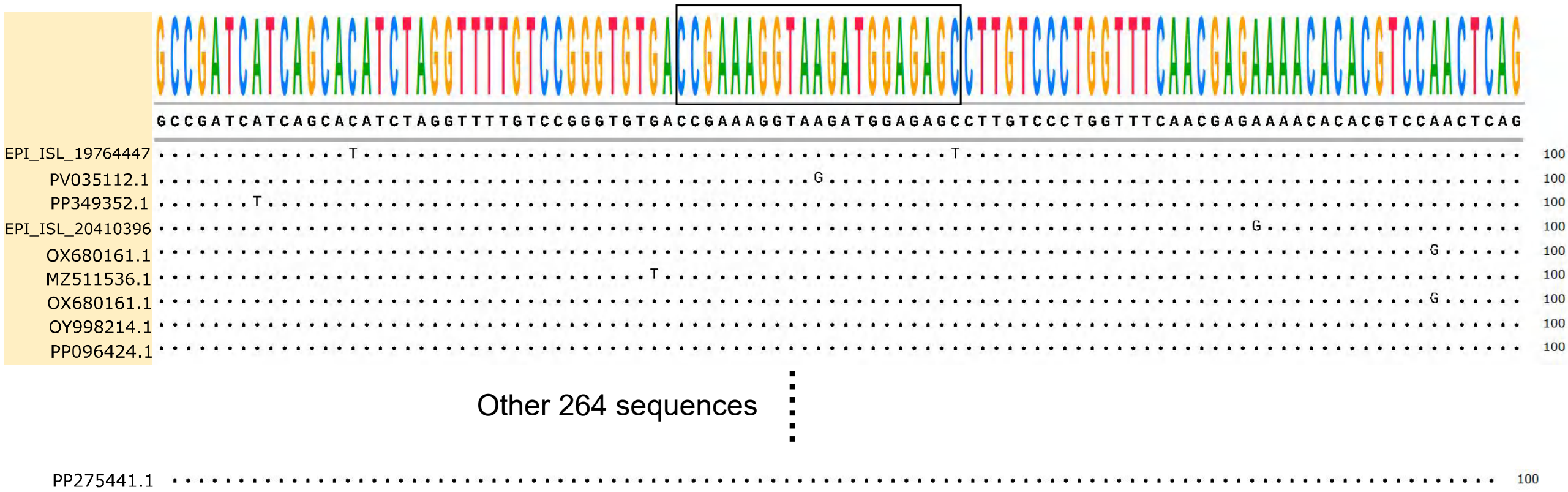

Fig. S1 Multiple sequence alignment of the ORF1ab ±50 bp region across 274 SARS-CoV-2 sequences in Fig 1b (except incomplete sequences). Dots indicate conserved nucleotide positions. The black box denotes the D-$(Arg)_8$-PNA_{21} target region.

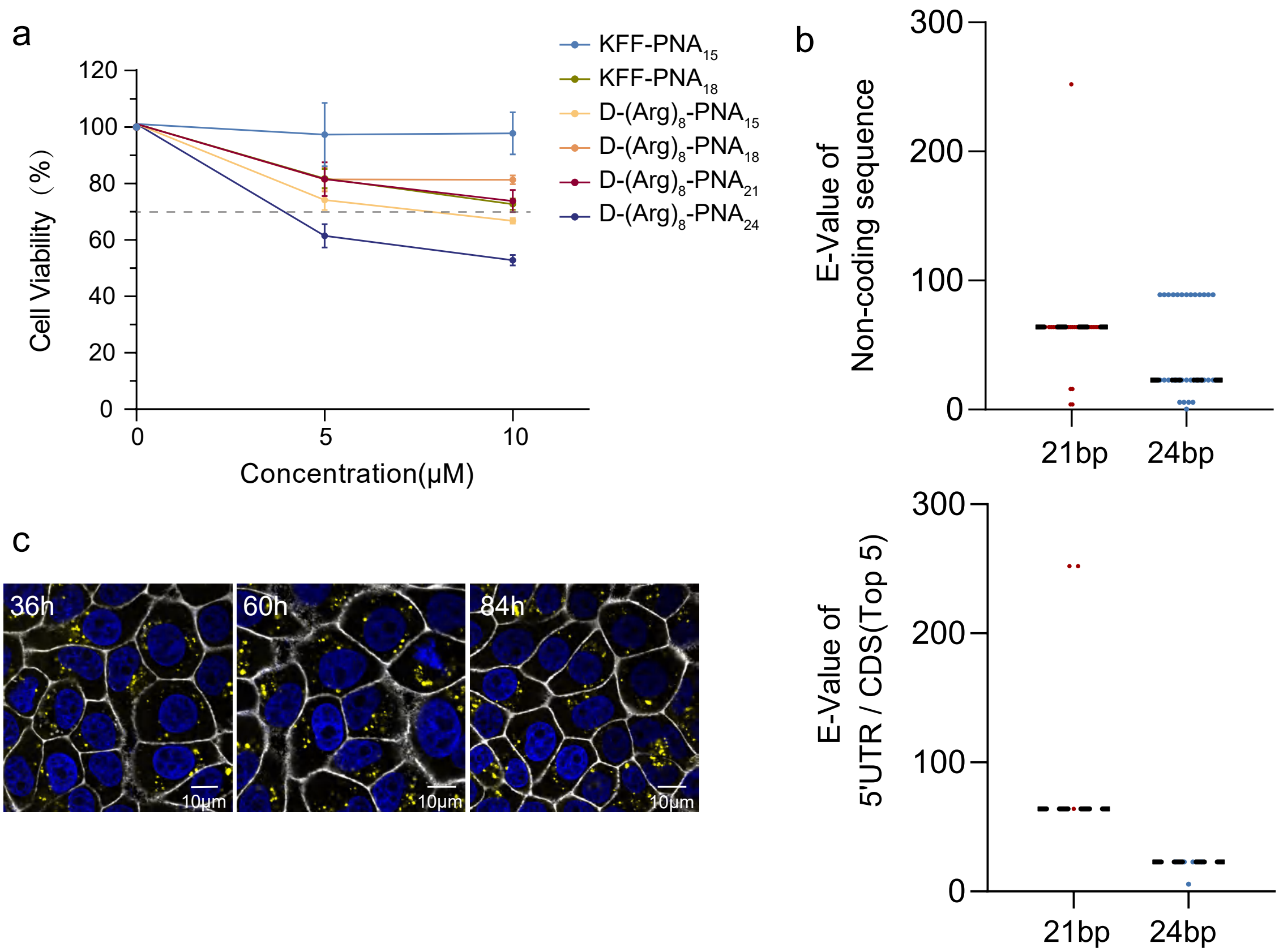

Fig. S2 Cytotoxicity and intracellular stability analysis of CPP-PNA sequences.

a. CCK-8 cytotoxicity assay of six CPP-PNA candidates in HeLa cells. Cells were treated with each sequence at 5 µM or 10 µM for 48 h. Following addition of CCK-8 solution, absorbance was measured at 450 nm using a microplate reader. Cell viability was calculated as the ratio of absorbance in each treatment group to that of the PBS-treated control. The dashed line at 70% indicates the threshold below which cytotoxicity was considered unacceptable. Data are mean ± SD (n = 3).

b. BLASTn analysis of D-(Arg)$_8$-PNA$_{21}$ and D-(Arg)$_8$-PNA$_{24}$ target sequences against the human reference RNA sequence database (refseq_rna). Search parameters are described in the Supplementary Methods. (Top) E-values for non-coding RNA transcript hits identified for each sequence. (Bottom) E-values of the top 5 hits mapping to the 5′UTR or CDS regions of human gene transcripts for each sequence. Black dashed lines indicate the median E-value.

c. Confocal microscopy images of HeLa cells treated with D-(Arg)$_8$-PNA$_{21}$-Cy5, acquired at 36, 60, and 84 hours post-treatment. Cell membranes were labeled with Alexa Fluor 488-WGA (shown in white pseudocolor) and nuclei were counterstained with DAPI (blue). Scale bar: 10µ m.

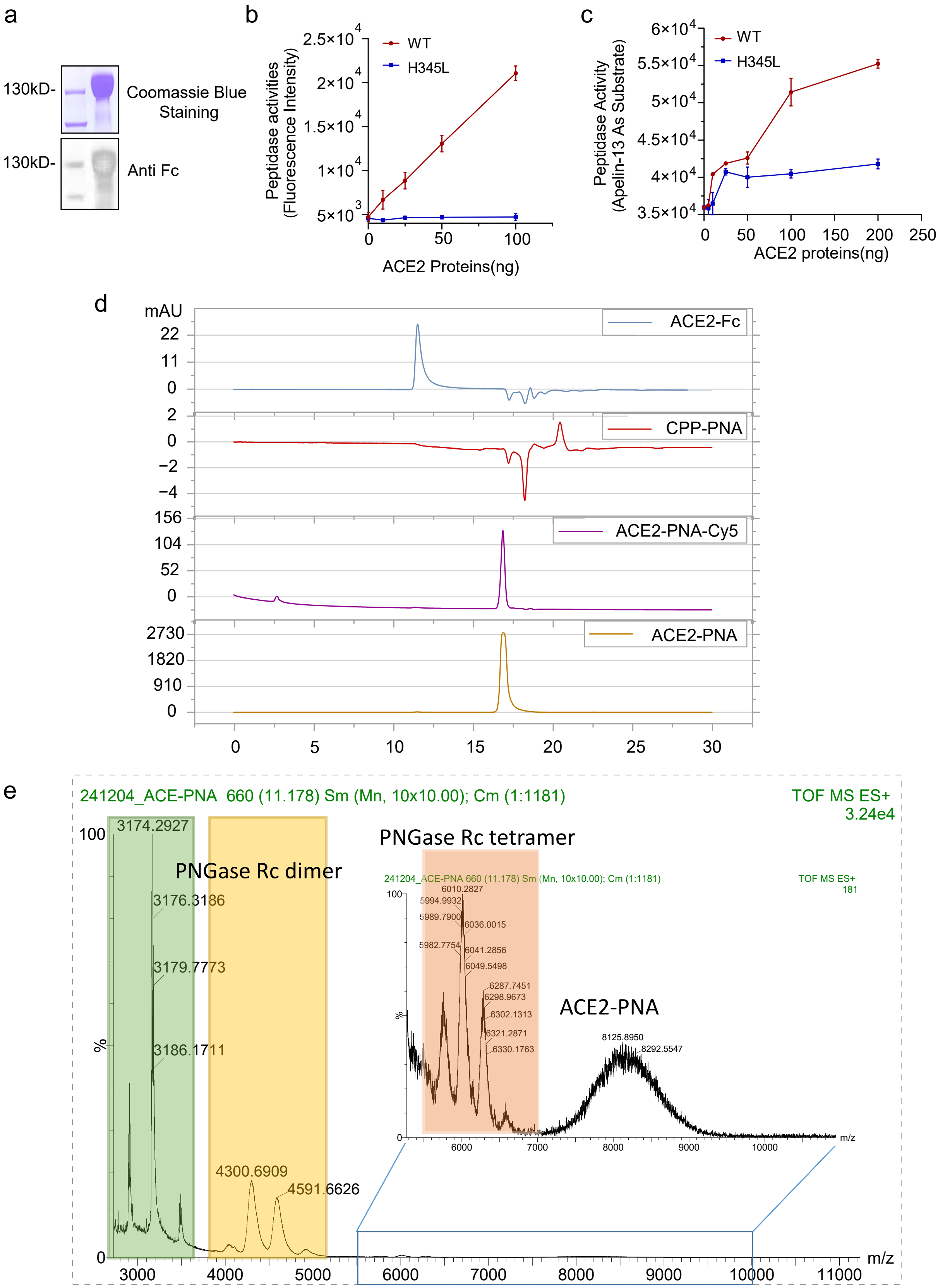

a
130kD-
Coomassie Blue Staining
130kD-
Anti Fc
b
WT
H345L
Peptidase activities (Fluorescence Intensity)
ACE2 Proteins(ng)
c
WT
H345L
Peptidase Activity (Apelin-13 As Substrate)
ACE2 proteins(ng)
d
mAU
ACE2-Fc
CPP-PNA
ACE2-PNA-Cy5
ACE2-PNA
e
241204_ACE-PNA 660 (11.178) Sm (Mn, 10x10.00); Cm (1:1181)
TOF MS ES+
3.24e4
PNGase Rc dimer
PNGase Rc tetramer
ACE2-PNA
3174.2927
3176.3186
3179.7773
3186.1711
4300.6909
4591.6626
6010.2827
5994.9932
5989.7900
6036.0015
5982.7754
6041.2856
6049.5498
6287.7451
6298.9673
6302.1313
6321.2871
6330.1763
8125.8950
8292.5547
m/z

Fig.S3 Characterization of ACE2-Fc mutant protein and ACE2–PNA conjugate.
a. SDS-PAGE analysis of ACE2(H345L/1-740)-Fc protein. The construct was expressed in HEK293F cells and purified via Fc-tag affinity chromatography. Protein integrity and purity were confirmed by Coomassie brilliant blue staining and western blot.
b-c. Carboxypeptidase activity assays confirming complete abolishment of enzymatic activity in ACE2-Fc(H345L) using the surrogate fluorescent substrate Mca-APK(Dnp) (b) and the physiological substrate Apelin-13(c). WT means wild-type ACE2-Fc protein, H345L means ACE2-Fc(H345L) mutant. Data are mean ± SD (n = 3).
d. Reverse-phase HPLC chromatogram of the ACE2-PNA reaction mixture. The ACE2-PNA or ACE2-PNA-Cy5 conjugate displayed a dominant, symmetric peak at approximately 16.9 min, while ACE2-Fc eluted at approximately 11.5 min, and CPP-PNA eluted after 20min.
e. Native mass spectrometry (nMS) analysis of the ACE2–PNA conjugate. Prior to analysis, N-linked glycans were removed by PNGase F treatment at 37°C for 4 h. The spectrum displays a broad charge-state envelope centered at approximately m/z 8,000, consistent with previously reported nMS data for ACE2. Owing to the difficulty in resolving individual charge states, the precise conjugation ratio between ACE2-Fc and PNA could not be determined.

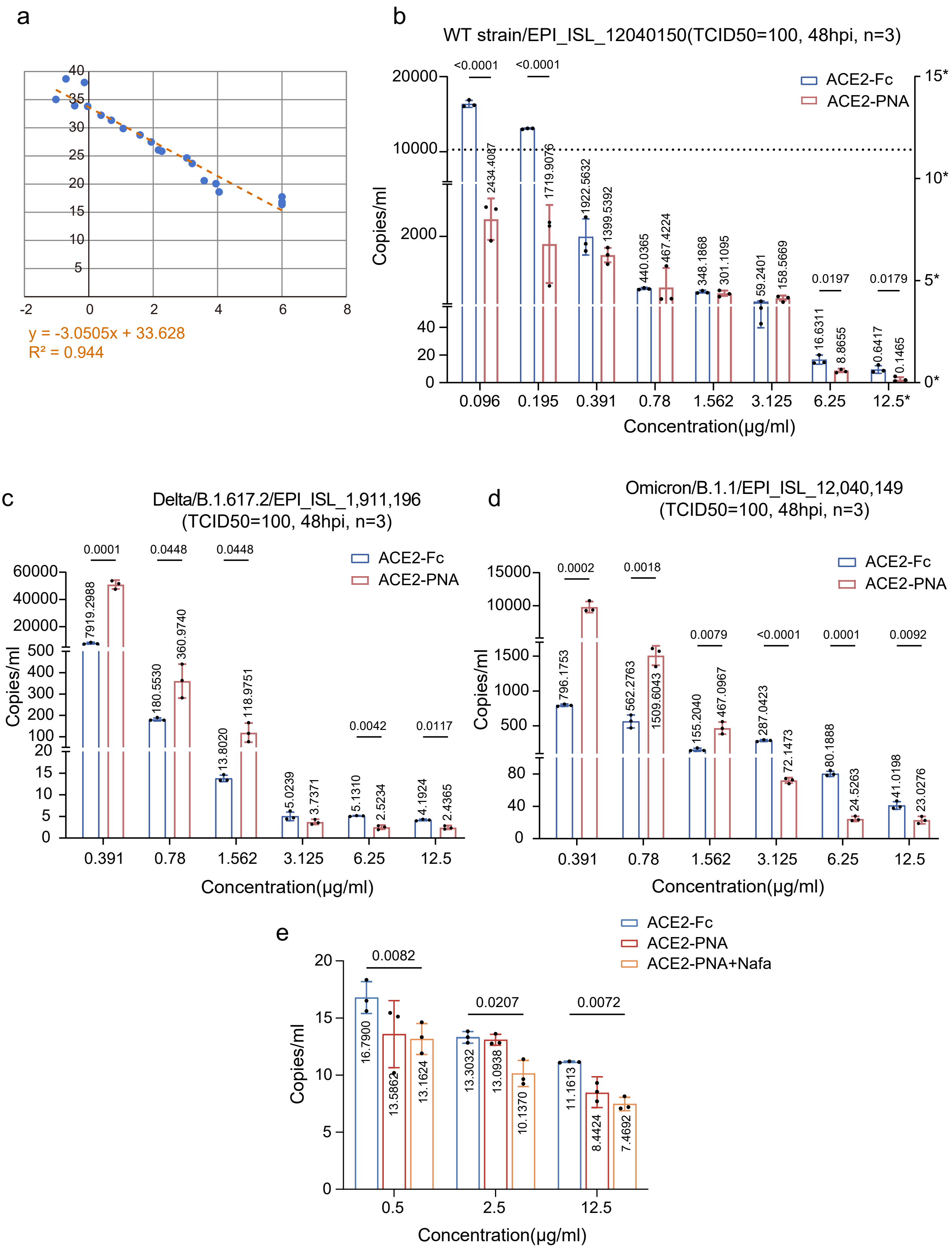

a
y = -3.0505x + 33.628
R² = 0.944
b
WT strain/EPI_ISL_12040150(TCID50=100, 48hpi, n=3)
ACE2-Fc
ACE2-PNA
Copies/ml
Concentration(μg/ml)
c
Delta/B.1.617.2/EPI_ISL_1,911,196
(TCID50=100, 48hpi, n=3)
ACE2-Fc
ACE2-PNA
Copies/ml
Concentration(μg/ml)
d
Omicron/B.1.1/EPI_ISL_12,040,149
(TCID50=100, 48hpi, n=3)
ACE2-Fc
ACE2-PNA
Copies/ml
Concentration(μg/ml)
e
ACE2-Fc
ACE2-PNA
ACE2-PNA+Nafa
Copies/ml
Concentration(μg/ml)

Fig. S4 Quantification of Absolute Viral Copy Numbers in Live Virus Experiments by Droplet Digital PCR (ddPCR).

a. Standard curve correlating the Ct values in qRT-PCR with absolute viral copy numbers per unit volume. RNA extracted from Vero E6 cell culture supernatants was serially diluted and subjected in parallel to qRT-PCR and ddPCR. The X-axis represents log10(copies/mL) and the Y -axis represents Ct values. Linear regression of the scatter plot yielded the standard curve: Ct=(-3.0505)×log10(copies/mL)+33.628. As this calibration was performed in an independent experiment, absolute viral copy numbers derived from this standard curve are reported as reference values.

b-e. Absolute viral copy numbers remaining in culture supernatants at each treatment concentration of ACE2-Fc and ACE2-PNA. (b)wild-type/ EPI_ISL_12040150; (c)Delta/B.1.617.2/EPI_ISL_1911196 in Vero E6; (d)Omicron/B.1.1.529/EPI_ISL_12040149; (e)Delta/B.1.617.2/EPI_ISL_1911196 in Calu-3. Specially, values for the 12.5 μg/mL treatment group (indicated by an asterisk) in (b) correspond to the right Y-axis. Data are mean±SD (n = 3). Statistical significance was determined by multiple unpaired Student's t-tests. Only comparisons with P ≤ 0.05 are indicated with connecting lines and values on the figure.

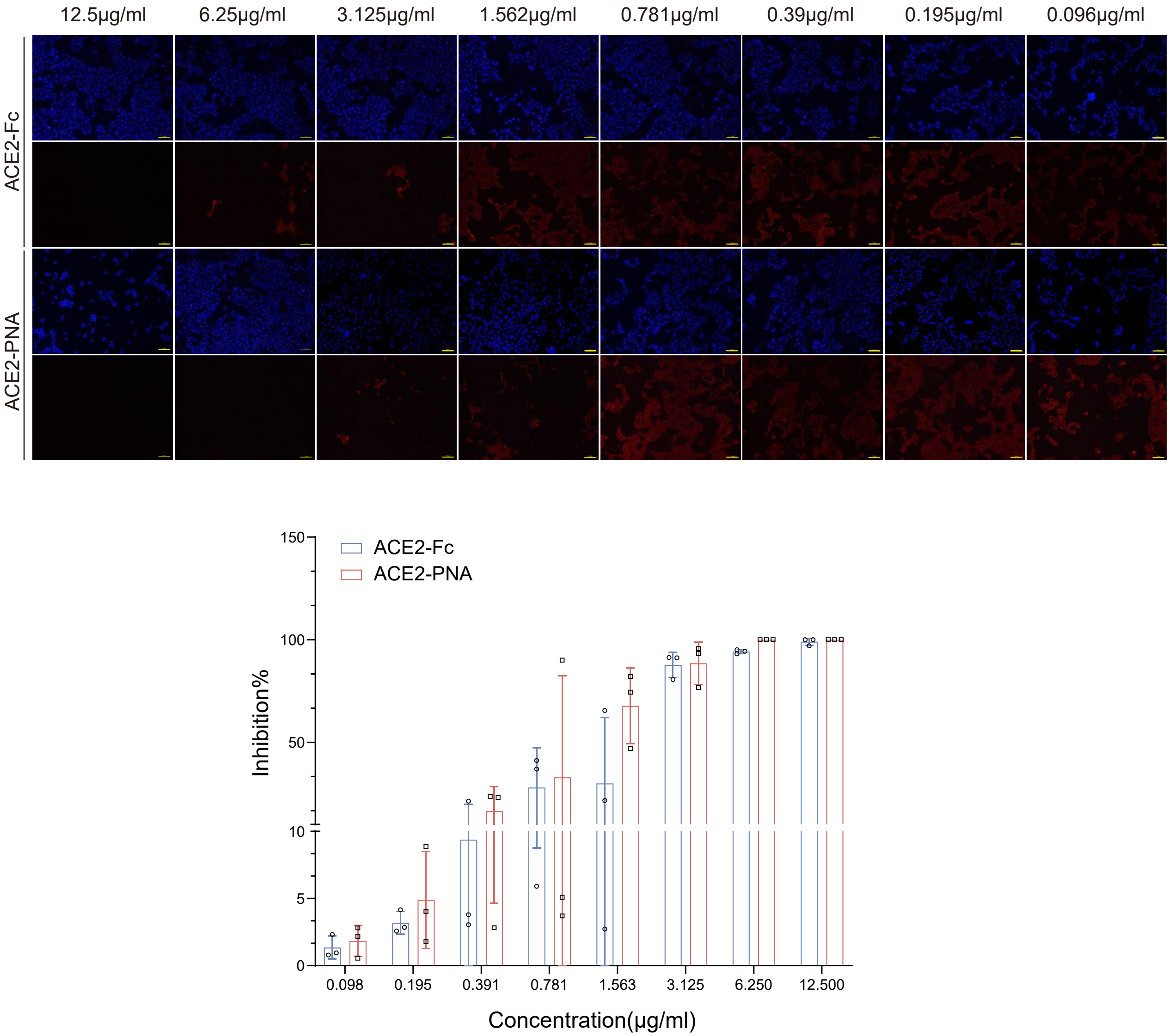

Fig.S5 Immunofluorescence images and quantification of Vero E6 cells infected with SARS-CoV-2 WT (EPI_ISL_12040150) under ACE2-Fc or ACE2-PNA treatment. Scale bar: 100μm. Quantification was performed using ImageJ by automated signal detection and manual cell counting. Date are mean ± SD (n = 3).

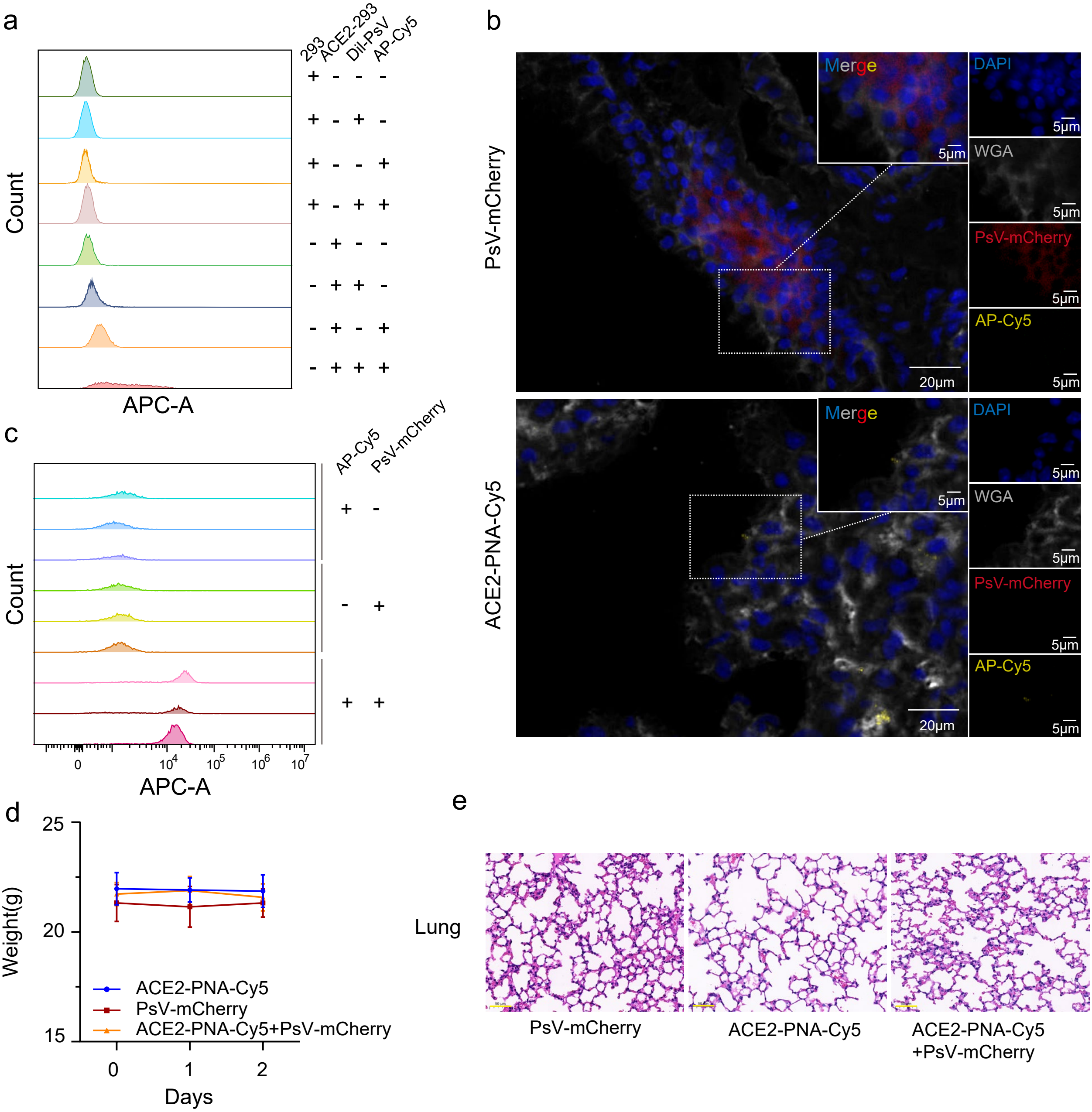

Fig. S6 Supplementary data from pseudovirus infection experiments in cells and mice.

a. Flow cytometric histograms corresponding to the data presented in Fig. 4a. ACE2-PNA-Cy5 was shorten as AP-Cy5.

b. Typical confocol images of lung cryosections from K18-hACE2 mice administered PsV-mCherry or ACE2-PNA-Cy5 alone, serving as single-treatment controls. Cell membranes and nuclei were labeled with Alexa Fluor 488-WGA and DAPI respectively. Scale bar: 20 µm.

c. Flow cytometric histograms corresponding to the data presented in Fig. 5c.

d. Daily body weight measurements of mice following administration of the indicated treatments.

e. Typical images of hematoxylin and eosin (H&E)-stained lung sections. Scale bar: 50µm.
